# X-ray Computed Tomography Analysis of Historical Woodwind Instruments of the Late Eighteenth Century

**DOI:** 10.3390/jimaging8100260

**Published:** 2022-09-24

**Authors:** Francesca Tansella, Luisa Vigorelli, Gabriele Ricchiardi, Alessandro Re, Letizia Bonizzoni, Sabrina Grassini, Manuel Staropoli, Alessandro Lo Giudice

**Affiliations:** 1 Dipartimento di Fisica, Università degli Studi di Torino, Via Pietro Giuria 1, 10125 Torino, Italy; 2 Dipartimento di Fisica “Aldo Pontremoli”, Università degli Studi di Milano, Via Celoria 16, 20133 Milano, Italy; 3 INFN, Sezione di Torino, Via Pietro Giuria 1, 10125 Torino, Italy; 4 Dipartimento di Elettronica e Telecomunicazioni, Politecnico di Torino, C.so Duca degli Abruzzi, 24, 10129 Torino, Italy; 5 Dipartimento di Chimica, Università degli Studi di Torino, Via Pietro Giuria 7, 10125 Torino, Italy; 6 Dipartimento di Scienza Applicata e Tecnologia, Politecnico di Torino, C.so Duca degli Abruzzi, 24, 10129 Torino, Italy; 7 Conservatorio di Musica “Giuseppe Tartini”, Via Ghega, 12, 34132 Trieste, Italy

**Keywords:** cultural heritage, conservation, woodwind instruments, tomography, flute

## Abstract

In this work, two historical flutes of the late eighteenth century were analysed by means of X-ray computed tomography (CT). The first one is a piccolo flute whose manufacturer is unknown, though some features could suggest an English or American origin. The second musical instrument is a baroque transverse flute, probably produced by Lorenzo Cerino, an Italian instrument maker active in Turin (Italy) in the late eighteenth century. Analyses carried out provided information on manufacturing techniques, materials and conservation state, and are suitable to plan restoration intervention. In particular, through the CT images, it was possible to observe the presence of defects, cracks, fractures and previous restorations, as well as indications of the tools used in the making of the instruments. Particular attention was directed towards extracting metrological information about the objects. In fact, this work is the first step of a study with a final aim of determining an operative protocol to enable the making of precise-sounding copies of ancient instruments starting from CT images, that can be used to plan a virtual restoration, consisting in the creation of digitally restored copies with a 3D printer.

## 1. Introduction

Most of the historical musical instruments in museum collections were designed as functioning objects, meant to communicate, impress, and entertain. Museum curators have the role of preserving and transmitting the materials and the intangible characteristics of these objects. 

For this reason, most museums do not allow historical wind instruments to be played: this conservative policy is due to the humidity produced by the musician’s breath during a performance and consequently absorbed by the instrument itself. Variations in humidity and temperature can cause irreversible damage and permanent distortions, which would irremediably alter the sound of the instrument. In this conservation strategy, adopted to preserve instruments’ integrity, there is an intrinsic contradiction: to preserve their original sound, they are made silent, depriving them of their musical meaning. The need to preserve historical musical instruments in terms of sound, is also due to the fact that a historical piece of music can sound different on modern instruments compared to the ones used at the time in which the piece was written [1]. 

In the past, this problem has been partially overcome by asking expert craftsmen to create replicas based on manual measurements of the original specimens. The degree of precision and, therefore, compatibility obtained between the original and the copy, however, is not yet sufficiently high, given the difficulties both in carrying out precise measurements and in the manufacturing of the copy.

The possibility of using non-invasive analysis techniques and digital technologies, such as computed tomography and 3D printing, for measurement and subsequent modelling, offers new opportunities for the reproduction of historical wind instruments and thus to restore their forgotten sound, as for example in [2], despite the work being limited to copying a small portion of an instrument.

X-ray Computed Tomography (CT) scanning is an analytical technique already successfully applied to the study of the internal structure of different types of cultural heritage objects [3,4,5,6]. In the field of musical instruments, the complexity of the shapes, the variety of materials used and the size of the objects belonging to this category make the acquisition of high-quality CT more complex. The process of acquiring meaningful CT of musical instruments has tackled by previous projects, and detailed technical guidelines have been published [7]. However, we have noted that the adoption of these guidelines is not sufficient. When the purpose of the tomography is the unique (and possibly automated) extraction of accurate digital geometrical models for the creation of acoustically faithful copies, further metrological and practical specifications are needed. It is important to define and fix all the analysis parameters (geometry, energy, scan time, etc.) before the experiment and, during the data analysis, not to underestimate the presence of possible artifacts, which could affect both the visual interpretation and the final modelling of the original object. 

Prior to this work, CT scans of musical instruments were already performed. The main objectives of these studies were mostly diagnostic, mainly for the state of conservation assessment, or with the purpose of showing and studying the internal structure of the instruments [8,9,10,11,12,13,14].

Since the first studies carried out in 1997 by Sirr and Waddle, using a CT medical instrumentation, several authors have described the use of the tomographic technique for the identification of internal damage or any previous restoration operations and for the determination of the authenticity in high quality instruments, demonstrating the potential of the technique.

The innovation of the analysis presented in this paper lies in the goal of the study: starting from indications provided by the previous MUSICES project [7], two flutes of the late eighteenth century were analysed, in order to develop an operational protocol for the creation, using a 3D printer, of acoustically faithful copies of the originals. This aspect is supported by the fact that the sound of low-pressure wind instruments is determined to the largest extent by the shape of the oscillating air cavity, with very limited effect of the material comprising the instrument. This is the subject of a long and dividing debate amount scientists and musicians, the former denying a significant effect of the material (see e.g., [15]) and the latter claiming to be able to appreciate perceptible differences even among instruments made of different wood species. The discussion has recently been found a consensus, at least concerning the wood instruments, with acoustical studies demonstrating the effect of the surface structure of different woods on the sound of resonating tubes [16].

This work illustrates the results regarding the conservation status of the analysed objects, and can be used both to plan a restoration intervention on the originals and to create a “virtual” repair, thus arriving at the creation of digitally and then physically restored playable copies.

## 2. Materials and Methods

### 2.1. Historical Woodwind Instruments

**Figure 1:**
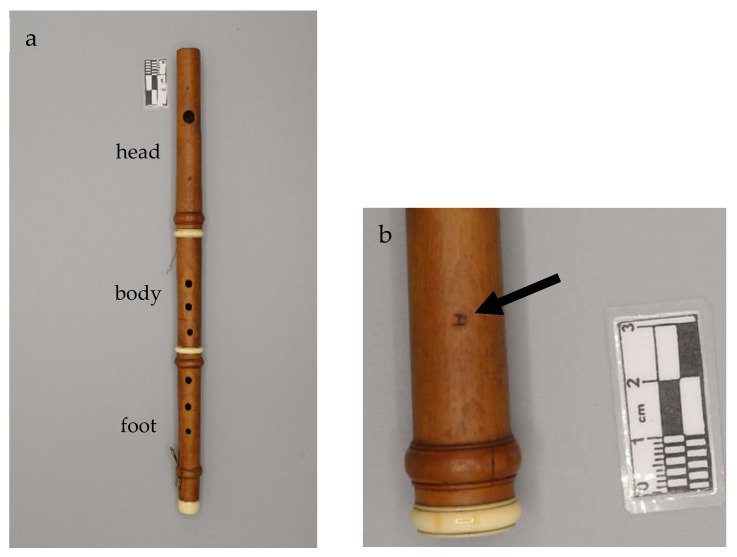
The analyzed woodwind instrument: (**a**) Piccolo Flute (tone in D), anonymous maker; (**b**) detail of the Piccolo Flute head, with the “D” marking (black arrow).

The objects of this study are two historical woodwind instruments belonging to a private collection. The first one is here designated as the Piccolo Flute (Figure 1), whose manufacturer is unknown; the only mark present is a “D” indicating the tuning of the instrument [17]. This flute is made of boxwood and is characterized by the presence of three ivory rings. Unlike the majority of the European historical piccolo flutes, composed of two or four parts, it consists of three parts: the head, the central body and the foot with a brass key. In fact, a quick survey of all the piccolo flutes present in the MIMO Database [18] yields only 11 three-part instruments out of about 250 items. This observation also helps in the dating of the instrument, since all the three-part instruments are dated between the last years of the 18th century and the beginning of the 19th. Furthermore, the presence of a larger mouth hole, compared to the specimens of the same period, suggests that the instrument might have been subsequently modified to be adapted to the taste and the higher standard pitch of the nineteenth century. 

**Figure 2:**
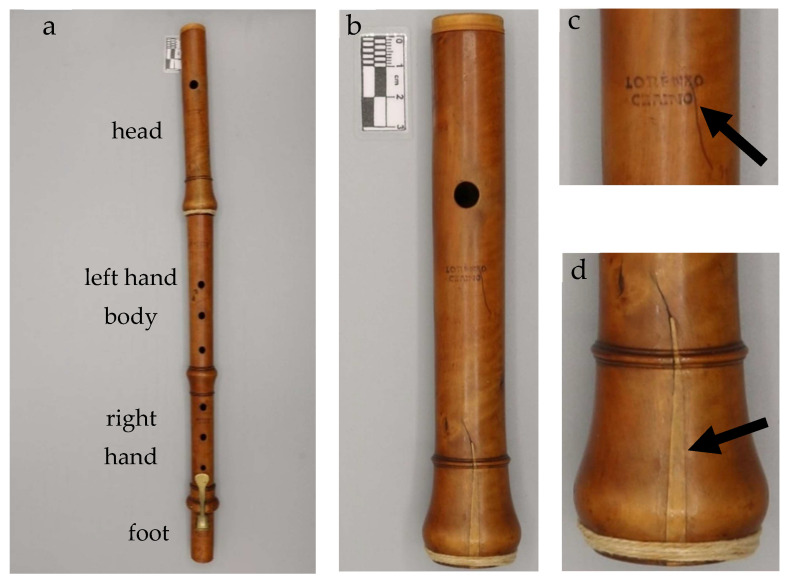
The analyzed woodwind instrument: (**a**) Transverse Flute (tone in F) produced by Lorenzo Cerino; (**b**) detail of the Transverse Flute head, in which it is possible to observe the manufacturer’s signature ((**c**), black arrow) and the crack ((**d**), black arrow).

The second object of this study is a baroque transverse flute, also known as “traversiere” (in F) belonging to the same private collection, probably produced by Lorenzo Cerino, an Italian instrument maker active in Turin in the late eighteenth century [19]. The authorship is suggested by the clear signature present on all the pieces of the flute (Figure 2). This instrument, here designated as the Transverse Flute, is made of boxwood and has a very traditional construction. It consists of four parts: a head with a modern cap (the original one has been lost); two central bodies, characterized by the presence of six holes and joined by mortise and tenon joints, sealed with waxed cotton yarn; and a foot with a brass key. The key is kept closed by a metal spring riveted to the lower face of the key. The tightness of the key is guaranteed by a thin layer of cork covered with a piece of soft leather.

At first sight some features about the state of conservation can be noticed. The head is characterized by a crack restored with the insertion of a wooden wedge, with a cotton thread binding to avoid the spreading of the fracture (Figure 2d).

### 2.2. Experimental

**Figure 3:**
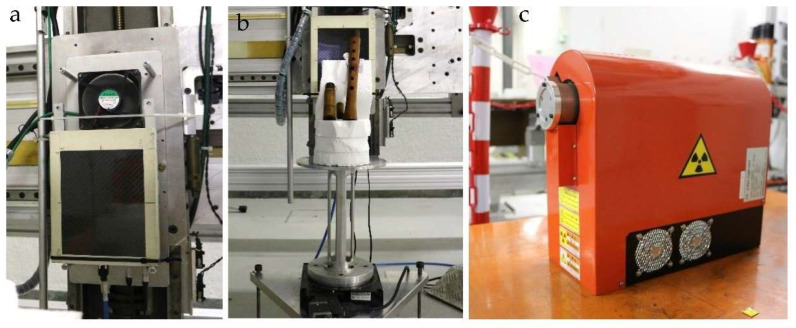
Experimental setup: (**a**) Flat-panel detector, (**b**) sample on the rotating platform, (**c**) X-ray source.

The tomographic analysis was carried out in a shielded laboratory for X-ray measurements at the Physics Department of the University of Turin. The instrumental apparatus was been specifically created using components already employed for other applications (Figure 3): a Hamamatsu Microfocus L8121-03 X-ray source, used, for example, for the micro-CT of pearls [20]; a rotating platform of about 20 cm in diameter on which the object under analysis is placed, allowing the acquisition of multi-angle images; a Flat Panel (FP) Shad-o-Box 6K HS detector, from Teledyne Dalsa, with a single pixel size of 49.5 μm and a 14-bit A/D converter, already used for the high-resolution CT of other wooden materials [21,22]. The main characteristics of the X-ray source and the flat panel are summarized in Table 1. The detector was installed on a mechanical X-Y scanning system that allows an optimal alignment with respect to the X-ray source. 

Since the detector has a sensitive area of 11.4 × 14.6 cm^2^, to scan larger areas it is necessary to acquire images in different positions by moving the object or the detector (tile scan). Another valid option is the use of the helical X-ray CT technique, which provides extra volume in the lengthways direction as the sample moves upwards along a helical path during the scan [9]. However, in our case, when the vertical extension of the object could be covered by a single scan, the data acquisition was divided into multiple scans, moving the object under analysis vertically. To allow the subsequent merging of the reconstructed volumes, avoiding the loss of information, the scans had to be carried out taking care to have a part of the volume overlapped. It is important that the overlapping region contains suitable reference points for the subsequent joining of the parts. If the analysed object does not have suitable elements, appropriate reference points could be introduced that make the arrangement of the different scans possible. In the recommendations deriving from the MUSICES project, the use of plastic beads inserted into an Ethafoam support is recommended. In our case, however, the holes of the flutes themselves were exploited. It was also decided to use the tips of toothpicks suitably fixed in the polystyrene support of the musical instruments. Both the instruments were disassembled for the CT analysis (the brass keys were also removed in order to avoid possible metal artefacts during reconstruction and volume segmentation phases), unlike what has been done in previous works [7], in order to ease subsequent segmentation of the tomography and to obtain better details of all the parts of the instruments. Due to the small size of the Piccolo Flute, compared with the active volume of the FP detector, it was not necessary to translate the object for tomographic acquisitions, differently from the Transverse Flute. CT analyses were performed by setting the integration time and the angular step on all 360° in order to optimize the quality of the final reconstruction. The main experimental details of this measurement are summarized in Table 2. 

**Figure 4:**
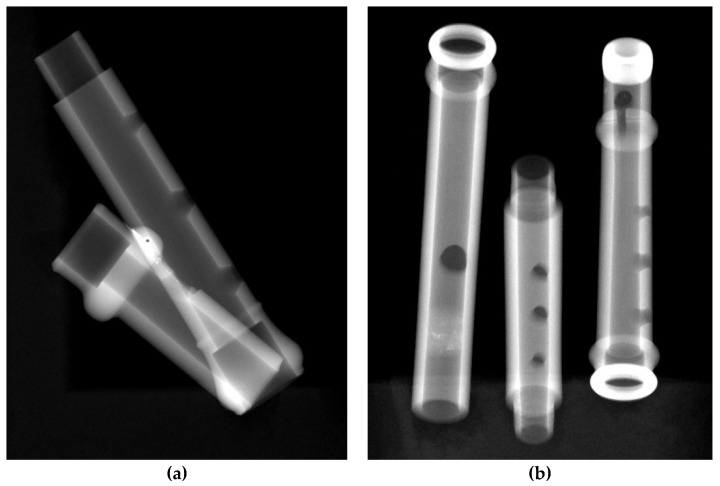
Normalized radiographs of the two analyzed instruments: (**a**) frontal view of the Piccolo Flute, (**b**) lateral view of the foot (without the brass key) and one of the two central bodies of the Transverse Flute.

After the CT acquisition of the object, additional images categorised as “white” (acquisition of projections without the object with the source turned on to directly record the distribution intensity of the X-ray beam) and “dark” (projections with the source turned off to record the background noise of the detector) were obtained. This procedure is fundamental for the subsequent elaboration of the acquired raw data, in order to exclude the background noise and normalize both for the beam inhomogeneity and the detector pixel response. This step of data processing consists of using the following formula (from the Lambert Beer law [23]) for normalization:

(1) $$I_{norm}=-\ln\frac{I_{raw}-D}{W-D}$$

where *D* stands for dark (background noise to be subtracted) and *W* stands for white (X-ray beam distribution intensity correction). In this case, white and dark images are both the average of 20 recorded projections. Following these operations, in the images obtained (called “attenuated radiographs”), the signal is proportional to the attenuation coefficient of the materials in the sample and the thickness crossed (Figure 4).

The subsequent reconstruction of the CT sections was carried out using a FDK (Feldkamp, Davis and Kress) algorithm (cone-beam geometry, [24]) by means of Dragonfly (version 2022.1, ORS Inc., Montréal, QC, Canada) a commercial software-utility [25], with which the subsequent 3D rendering was also performed. After the reconstruction, the Dragonfly software allows the observation and study of the reconstructed three-dimensional volume and of the sample sections along the three axes of the Cartesian plane.

At this point, if the sample has been acquired in several sections, it is necessary to stitch them up, using precise common points as a reference.

It must be highlighted that information from CT is full of internal details of the object and is very useful for its physical analysis, but it is not directly usable to make a copy with 3D printing techniques, which is the final aim of this study. In fact, the structure of the wood is of some interest for the reconstruction of copies of the flute, because it determines the anisotropy of the mechanical properties as well as the ovalisation of circular sections upon aging. The former has been neglected in this study both because of the very limited influence on sound and because the 3D printing technique we chose for reproduction does not allow the control of anisotropy. The latter (ovalisation) appears to be very limited and in our geometrical models all of the defects and effects of ageing on the shape of the instrument were included. A description of the object surface is required for the subsequent 3D printing step. It is therefore necessary to extract a geometric model of the wood surface from the tomography and export it as a mesh (using the Dragonfly software, the same used for the reconstruction cited above), carrying out a virtual restoration and excluding all the internal details of the object (veins, cavities, density variations, …).

## 3. Results and Discussion

**Figure 5:**
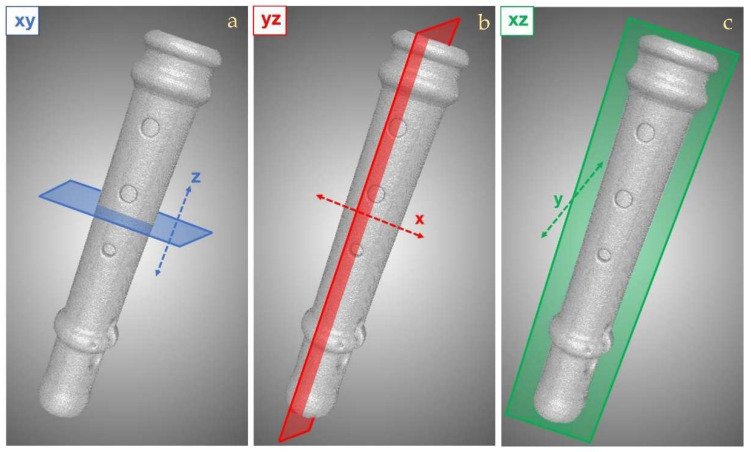
Schematic representation of the three CT reconstructed slices direction: (**a**) transversal or horizontal sections correspond to the xy plane along the z axis; (**b**) sagittal or lateral sections correspond to the xz plane along the y direction; (**c**) longitudinal or vertical sections correspond to the yz plane along the x direction.

The performed analysis allowed some qualitative and first quantitative observations on the state of conservation of the analyzed samples. Thanks to computed tomography it was possible to highlight, apart from the typical wood structures, i.e., growing rings, the presence of:

- fractures,
- cracks associated with wood knots,
- holes and manufacturing defects in the bore,
- areas with higher radiopacity (high density material inclusions),
- fracture repairs,

Below, some CT reconstructed slices of the two investigated instruments show the main observations. For greater clarity, the three spatial directions of the reconstructed tomographic sections are displayed in Figure 5, with the relative planes that will be reported in all the figures shown afterwards.

### 3.1. Piccolo Flute

**Figure 6:**
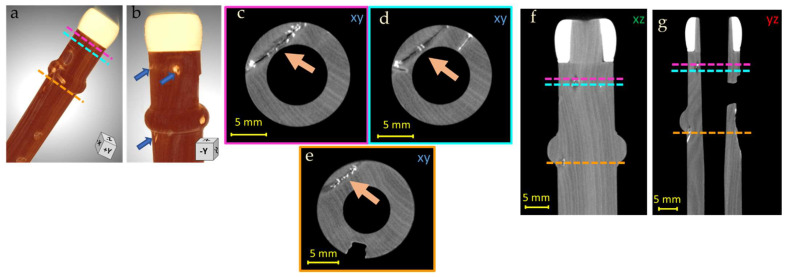
3D render of the Piccolo Flute foot (**a**,**b**) with the indication of the illustrated features in the following pictures: CT horizontal (**c**–**e**), vertical (**f**) and lateral (**g**), slices of the Piccolo Flute, in which the fractures originating from the knots and their restoration are visible (orange arrows); in (**f**,**g**) colored lines correspond to the different fractures at different heights (colored contours of (**c**–**e**)).

The Piccolo Flute has in general a good state of conservation; however, some issues were observed. In the foot of the instrument, some cracks in correspondence with wood knots can be seen clearly from the reconstructed CT slices in the three different directions (Figure 6). In particular, from the horizontal section (Figure 6c–e) it is possible to observe that the fractures cross the entire internal part of the wood in a transverse direction (9.3 mm for the one in the upper part and 8 mm for the second one) and to appreciate the presence of a different material in correspondence to the fractures. The high X-ray opacity of this material, partially filling the gaps, suggests masking or restoration interventions carried out in the past, probably with a metal-containing filler, to avoid the propagation of the fractures.

**Figure 7:**
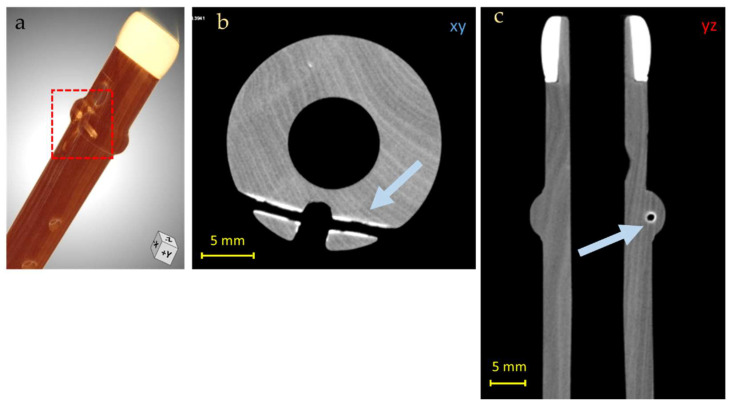
3D render of the Piccolo Flute foot (**a**) and CT horizontal (**b**) and lateral (**c**) slices of the Piccolo Flute foot, in which the supposed metallic residues from the brass key insertion is observed (blue arrows in (**b**,**c**) and red square in (**a**)).

In addition, the presence of a radiopaque area was observed in correspondence to the brass key insertion area and all around the hole where a metal pin used to ensure the key tightness was placed (in the foot of the instrument, Figure 7), probably due to the absorption of metal ions by the wood, generated by the oxidation of the brass.

**Figure 8:**
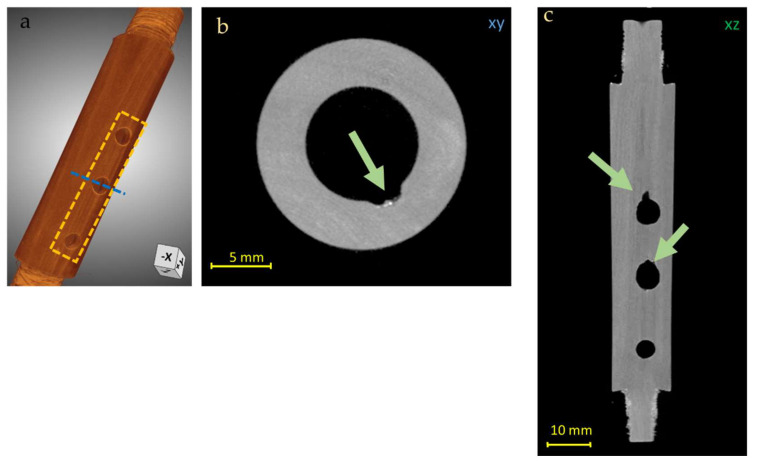
3D render of the Piccolo Flute body (**a**) and CT horizontal (**b**) and vertical (**c**) slices of the Piccolo Flute body, in which imperfections in the holes are observed (green arrows in (**b**,**c**), yellow square in (**a**)). Blue line in (**a**) indicates the high of the XY slice (**b**).

In the central body of the instrument, some imperfections in correspondence to the first and the second holes can be observed (Figure 8), especially from the lateral slice (xz plane, Figure 8b); some possible explanations of these features could be that this damage was produced during a careless restoration intervention or, as is often the case for historical woodwinds, in an attempt to raise the pitch by enlarging the tone holes.

**Figure 9:**
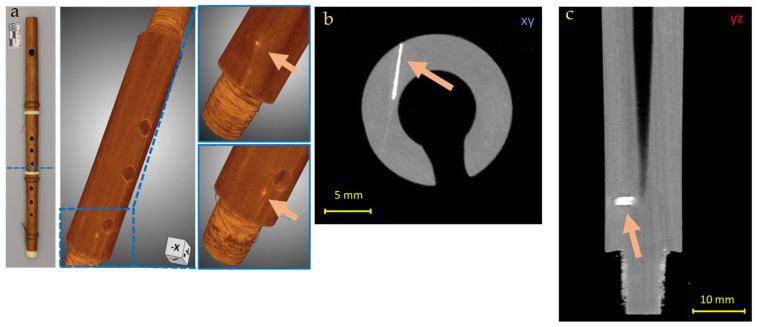
Original and 3D render of the Piccolo Flute body (**a**) and CT horizontal (**b**) and lateral (**c**) slices of the Piccolo Flute body, in which denser filling material is clearly visible inside the wood (orange arrows in (**b**,**c**)). In (**a**) the areas in which the denser material is present (blue line and square) are highlighted.

Another particular feature was observed in correspondence to the bottom part of the body: from the horizontal and vertical slices, a more radiopaque material is perfectly visible inside the wood structure (Figure 9). This could be a denser filling material used for a fracture reparation, that extended from the external surface of the instrument to the internal part for 6.3 mm in the transversal direction (Figure 9a) and 1.4 in depth (Figure 9b).

### 3.2. Transverse Flute

The greatest damage was observed on the Transverse Flute, in three of the four pieces into which it is divided.

**Figure 10:**
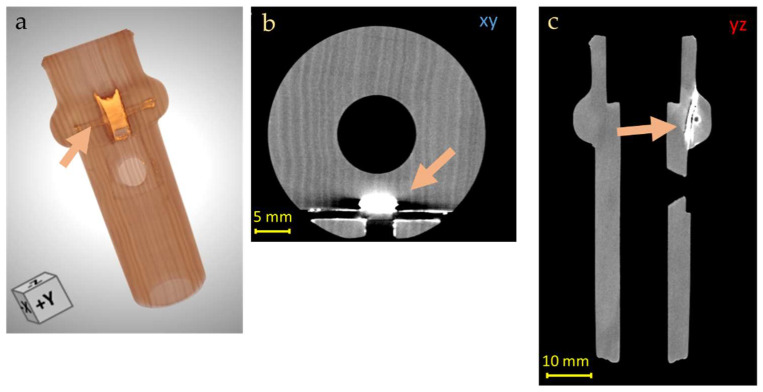
3D render (**a**) and CT horizontal (**b**) and lateral (**c**) slices of the Transverse Flute foot, in which the metallic residues from the brass key insertion are observed (orange arrows). In the 3D rendering (**a**), the metallic residues are also clearly visible in the upper part of the model.

As for the Piccolo Flute, in the terminal part of the instrument (the foot), it is possible to observe the absorption of metal ions where the brass key is inserted. In this case, however, not only is the hole guiding the pin affected, but also the point of insertion of the spring (Figure 10).

**Figure 11:**
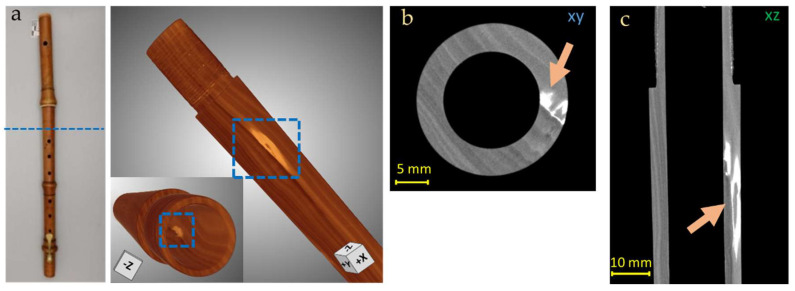
Original and 3D render (**a**) and CT horizontal (**b**) and vertical (**c**) slices of the Transverse Flute body (left hand), in which area of higher density was observed (orange arrows), also visible in the 3D model (blue squares). Blue line in the original pictures indicates the high of descripted feature.

At the level of the left-hand body, an area of higher density was observed, for which it is possible to hypothesize the presence of mineral inclusions in the wood or restoration interventions with mineral-based stucco (Figure 11).

**Figure 12:**
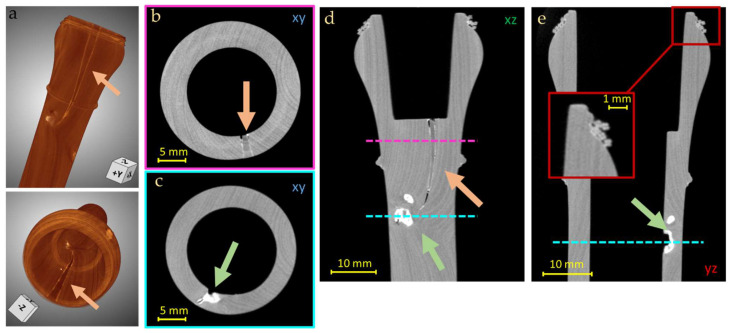
3D render (**a**) and CT horizontal (**b**,**c**), vertical (**d**) and lateral (**e**), slices of the Transverse Flute head, where the repaired cracks are visible (orange and green arrows) and a particular of the external cord fixing (**e**). The colored lines determine the height of the two damages base on the contour of (**b**,**c**).

The most significant damage, which impairs the sound of the instrument, is in the head of the Transverse Flute. The CT images show the crack extension (21 mm), already restored (Figure 12a–c, orange arrows). The repair of the crack present in the head with the insertion of a wooden wedge also explains the presence of a tight external cord along the circumference of the head itself (Figure 12d). Again, inside the bore of the head, the presence of more damage was highlighted together with an attempt to restore it, probably with putty, denser than the wood and thus with a higher grey level (Figure 12b–c, green arrows).

**Figure 13:**
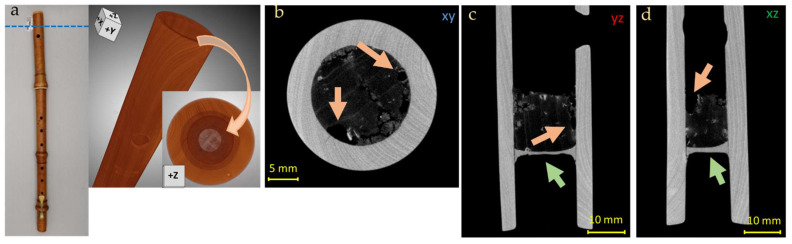
Original and 3D render (**a**) and CT horizontal (**b**), lateral (**c**) and vertical (**d**), slices of the Transverse Flute head, in which some voids in the cork (orange arrows) and the wax layer placed on (green arrow) are observed. Blue line in (**a**) indicates the height of the flute head in which the cork is inserted.

Finally, it was also possible to observe the state of conservation of the cork inside the instrument head, with an estimated size of 15 mm from the CT reconstruction. The position of the cork and its integrity is fundamental for the intonation of the Transverse Flute. From the analysis, non-optimal conservation conditions can be observed. In fact, it is no longer homogeneous, and has some relatively large areas lacking material (Figure 13). Lastly, it was possible to notice the presence of a thin wax layer (from 0.7 mm in the central thinner part to 3.5 mm in the thicker edge parts) placed on the cork. It has been hypothesized that this layer could be a restoration attempt, in order to remedy the non-tightness of the cork. Currently, this invasive method of restoration neither complies with present day standards for the conservation of musical instruments [26], nor does it make the instrument fully playable, because it limits its tunability.

## 4. Conclusions

The applications of X-ray computed tomography to cultural heritage allow non-destructive analysis of historical artifacts belonging to museums or private collections, where in most cases use of non-invasive diagnostic techniques is fundamental for their study. In this work, a particular class of objects was studied: two historical woodwind musical instruments were analysed by means of CT. Musical instruments in general are very interesting artifacts to study in order to obtain better knowledge of the manufacturing techniques, thereby discovering the ancient craftsmen’s secrets, and at the same time observing their state of conservation. Unfortunately, especially for woodwind instruments, it is no longer possible to play them, for conservation reasons, and thus it is not possible to hear their sound. We have presented the first steps of a wider project that involves CT analysis of two wind instruments, a piccolo flute and a transverse flute from the end of the eighteenth century, in order to finally create 3D digital models of the originals. Particularly in this paper, the observations made regarding the state of conservation of the analysed flutes permitted the evaluation of the presence of fractures, inner damage and past restorations. All of the information obtained can be used also to evaluate the possibility of new interventions on the original musical instruments. Furthermore, thanks to the possibility of creating a digital 3D model, CT scans can be used to make a “virtual” restoration, consisting in the creation of digitally restored copies with a 3D printer. A subsequent development of this study will include the creation of “sounding” copies of the original instruments, intended to be acoustically compatible with them, and tested with appropriate analytical methods. Furthermore, some dimensional evaluation of both digital models and physical copies will be performed, in order to ensure the compatibility and reach a more accurate understanding of all the protocol steps. 

## Figures and Tables

**Table 1:** Technical specifications of the equipment used.

Shad-O-Box 6K HS Flat Panel Detector		Hamamatsu Microfocus L8121-03 X-ray Source	
Pixel number	2304 × 2940	Target	Tungsten
Active area	11.4 × 14.6 cm^2^	Voltage	40–150 kV
Pixel size	49.5 μm	Max Current	500 μA
A/D converter	14 bit	Max Power	75 W
Energy range	15–225 keV	Focal Spot	Small ≈ 7 μm; Medium ≈ 20 μm; Large ≈ 50 μm
Scintillator	CsI	Beam angle	43°
Data transfer	Gigabit Ethernet	Exit window	Be, 200 μm

**Table 2:** Experimental CT conditions.

	Piccolo Flute	Transverse Flute
Dimension of the object	Length 339 mm	Length 427 mm
	Diameter~22 mm	Diameter~34 mm
Material	Boxwood, ivory	Boxwood
X-ray tube voltage	90 kV	
X-ray tube current	500 µA	
X-ray filter ^(a)^	Al (2 mm)	
Focal spot size	L (50 µm)	
Source-Detector Distance (SDD)	1400 mm	
Source-Object Distance (SOD)	1300 mm	
Object-Detector Distance (ODD)	100 mm	
Magnification	(1.08 ± 0.01)×	
Angular step	0.25°	
Integration time	4 s	
Number of projections	1440	
Scan phases (portions) ^(b)^	1	3
Total acquisition time ^(c)^	2 h 22 min	7 h 6 min
Reconstruct voxel size	(46 ± 2) μm	
Dimension of one CT scan	18.6 Gb	

^(a)^ The use of an aluminum filter avoids the beam hardening effect; ^(b)^ for objects higher than the detectable length more than one portion is acquired: here, the parameters for one portion are reported; ^(c)^ the total time is related to the total object (including the dead time due to the rotation stage movement and the image saving).

## Data Availability

The data presented in this article are available on request from the corresponding authors.
